# K-Means Clustering and Bidirectional Long- and Short-Term Neural Networks for Predicting Performance Degradation Trends of Built-In Relays in Meters

**DOI:** 10.3390/s22218149

**Published:** 2022-10-25

**Authors:** Jiayan Chen, Chaochun Zhong, Jing Chen, Yuanxun Han, Juan Zhou, Limin Wang

**Affiliations:** 1 College of Quality & Safety Engineering, China Jiliang University, Hangzhou 310018, China; 2 Zhejiang Key Laboratory of Energy Measurement and Environmental Protection, Zhejiang Province Institute of Metrology, Hangzhou 310018, China

**Keywords:** degradation trends, meter relays, neural networks, K-means clustering

## Abstract

The built-in relay in a meter is a key control component of a smart meter, and its reliability determines whether the user can use electricity safely and smoothly. In this paper, the degradation characteristics of the arc-burning energy are enhanced by the method of K-means clustering to replace degradation data, such as the overtravel time, release time, and other data. In existing methods, the meter needs to be disassembled to describe the degradation trend of the meter relay. The proposed method is combined with a bidirectional long short-term memory (Bi-LSTM) neural network to predict the degradation trend of the relay’s performance. In this paper, K-means clustering is used to enhance the extraction of arc energy data features, and then the arc energy data obtained from the reliability lifetime test is assessed to predict the degradation trend of the meter relay by means of a bidirectional LSTM.

## 1. Introduction

The meter relay, which is mainly used for remotely pulling and closing the meter, is an important part of the smart grid, and its position in the circuit directly affects whether the customer can use electricity normally. In general, the meter is not allowed to be pulled or closed under high currents to prevent the relay from corroding and sticking to the contacts, thus affecting normal opening and closing functions [1]. However, with the development of technology, relays are becoming increasingly reliable, and there are situations where higher loads and currents must be used. To protect all customers and ensure safety, the reliability of meter relays of pulling and closing under high current conditions must be taken into account.

Currently, reliability calculations are generally based on the number of failures of a product [2]; thus, the reliability of a product must be tested until the product fails. Alternatively, the reliability of a product is calculated by collecting degradation data during the timed reliability cutoff test [3]. However, with the increasing demand for reliable electronic products, the data obtained from the short-time cutoff test do not provide a good indication of the degradation trend, and the reliability calculated from the degradation data is often inaccurate [4]. In this paper, a Bi-LSTM neural-network-based method is proposed to extract features from the degradation data collected during the short-time cutoff test, and the subsequent degradation trend is predicted to calculate the accurate reliable life of the meter relay.

In previous methods of testing the reliability of relays, the relay must be removed from its original circuit, and the wires must be led out to measure the voltage and current inside the coil. The voltage and current data from the contacts are used to calculate the overtravel time or suction time, which is used to calculate the reliability of the relay [5]. However, metering devices are important, and thus, they are sealed and cannot be dismantled at will [6]. Therefore, a method for predicting the remaining life of a relay without breaking the seal of the meter by collecting only the contact currents and contact voltages is proposed in this paper.

There have been a few investigations on built-in relays in meters. However, for other types of relays or AC contactors, many scholars have proposed their own methods. Some scholars have proposed data-driven prediction models using various parameters, such as the overtravel time, contact resistance, bounce time, arc ignition time, and release time. Among these articles [7], a nonlinear Wiener process prediction model was developed to calculate the reliable life of a contactor using a third-order functional degradation model of contact mass loss and a time scale transformation model with a time scale transformation of the cumulative number of operations, using the first failure of the cumulative contact mass loss as the failure criterion. Research has also been carried out based on deep learning models in data-driven approaches; their highly adaptive nature has been applied to nonlinear data. In the literature [8], the mean impact value (MIV) algorithm was used for the problem of AC contactor life prediction, identifying the main inputs to the model as the cumulative arc ignition energy and suction time and predicting the reliable life of the contactor by means of an adaptive BP neural network model. In the article [9], a Bayesian approach to statistical analysis of overtravel time and bounce time was used to predict the relay contact performance through a method of continuously updating dynamic parameters. In the article [10], an LSTM model based on Harris hawks optimisation was developed for reliable life prediction by measuring the values of the contact resistance. However, metre relays, which have a characteristically high number of degraded layers of contact resistance with abrupt failure, are not suitable as characteristic parameters for long-time series prediction. In the article [11], several characteristic parameters, including suction time and overtravel time, were measured for prediction. However, only a small number of parameters that play a major role in the final prediction can be found in the results of this article. Arc-burning energy and overtravel time had the greatest impact on the life of the relay [12]. In most of these studies, the overtravel time is required as the main characteristic parameter to be substituted into the model, yet measuring the overtravel time would destroy the integrity of the meter. Moreover, none of these methods make good use of the fact that relay life is a long-time series.

**Figure 1:**
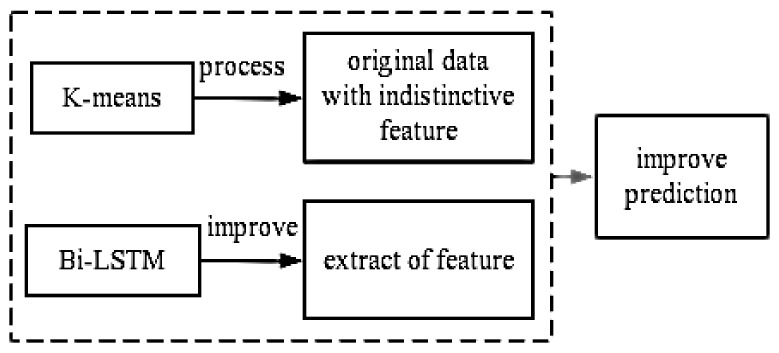
Methodological ideas.

In this paper, a key parameter for predicting the electrical life of a relay is designed to measure the arcing energy of the meter relay. The arcing energy is collected without disassembling the meter, i.e., it can be obtained from the main circuit. However, as the trend of the arc energy data over time is complex, it is difficult to extract the characteristic information, and inputting the data directly into the neural network would result in very unsatisfactory results. Therefore, K-mean clustering is used in this paper to determine the failure threshold of the relay based on the arc energy, which transforms the arc energy data and makes the characteristic information more obvious. The experimental results in this paper show that the trend of the arc energy data after transformation is not smooth and monotonic. As the LSTM neural network can only propagate in one direction, it can miss feature information when predicting its lifetime model. The Bi-LSTM network, on the other hand, is able to re-extract the feature information missed in the one-way propagation process to achieve more accurate model prediction. Therefore, a bidirectional long short-term memory (BI-LSTM) neural network is proposed in this paper to build a prediction model for the electrical life of meter relays. The ideas presented in this paper are shown in Figure 1 below. The results show that the proposed method can predict the degradation trend of the built-in relay of the meter with high accuracy while ensuring the integrity of the meter.

Therefore, this paper combines the K-means clustering and Bi-LSTM neural network methods to achieve the description of the degradation trend of the built-in relay of the energy meter through the arc-burning energy. The problem of describing the degradation trend of meter relays only by overtravel time and suction time is solved. Due to the fact that these parameters are more obvious with the increase of relay opening and closing times, but must disassemble meters to obtain them. The degradation parameter used in this paper, arc energy, can be collected directly in the main circuit of the meter.

The reasons for selecting arc energy as a feature parameter and its degradation mechanism are described in Section 2, and the basic principles of the model used in this method are described in Section 3. In Section 4, the reasons for using the K-means method to enhance feature extraction with specific data, its implementation in the transformation of the data, and how arc energy data were collected are described. In And the use of the Bi-LSTM approach to predict degradation trends in the transformed data is demonstrated, and the accuracy of the LSTM predictions is compared.

## 2. Material and Methods

### 2.1. Characteristics of the Research Subjects

The main characteristic parameters regarding relay reliability testing are the overtravel time, release time, suction time, and arc ignition time, which are calculated as shown in Table 1 below.

**Figure 2:**
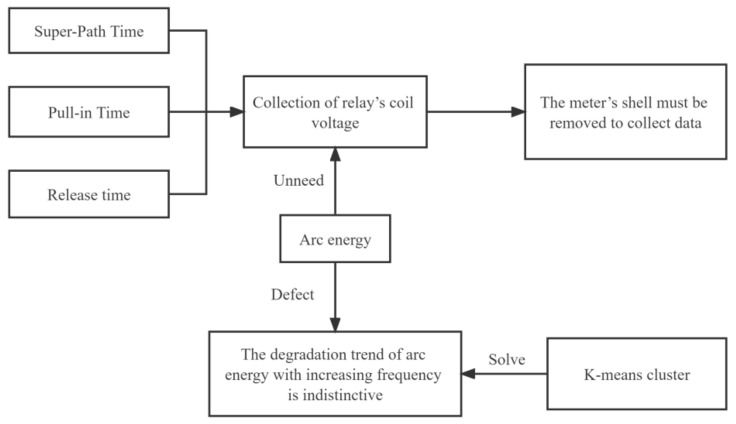
The reasons for selecting arc-burning energy as the input data.

In previous studies on the performance degradation of relays, the overtravel time and release time were generally selected as the main parameters to be studied because of their obvious degradation trends and the ease of extracting their features. The disadvantage is that the meter had to be dismantled [13,14,15] to measure the coil voltage of the relay, which is represented graphically in Figure 2. The arc-burning capacity is generally not chosen as the main degradation parameter because the trend of this function, i.e., the arc-burning energy with an increase in the number of openings and closings, is less obvious However, the acquisition of this parameter does not require dismantling the meter, and can be obtained only from the main circuit connected to the meter. In this paper, the K-means clustering method was used to change the expression of arcing energy, changing the relationship between the arcing energy and the number of openings and closings into a relationship between the frequency of occurrences above a certain energy threshold and the number of openings and closings. The degradation characteristic of the arc energy was successfully enhanced to yield a significant degradation trend.

In practical engineering applications [14], the generation of an arc is one of the main factors in the degradation of a relay. The main reason is that the high-temperature action of the arc melts the contact surface material, and the arc plasma takes molten droplets from the outlet surface and evaporates some of the substrate, resulting in a continuous loss of outlet material. In the case of a constant-contact wear mechanism, the greater the energy of the burning arc is, the greater the energy generated, and more arc plasma as well as heat melts the outgoing head material, resulting in a greater loss of contact mass [16]. Therefore, it is reasonable to choose arc energy as the performance degradation of the meter relay for this study.

### 2.2. Model Building

#### K-Means

K-means, as an unsupervised learning clustering algorithm, can achieve better results when dealing with numerical and unlabelled arc-burning energy data [17].

The steps for K-means clustering are as follows.

1. Input the sample set S = {$x_{1},x_{2},\ldots ,x_{m}$ }, which is the number of m-dimensional samples.
2. Calculate the maximum and minimum values in the sample set and record their occurrences as *a* and *b*, respectively. Calculate the results of rounding the mean of *a* and *b*, *c*, and then the initial clustering centre $\mu =\left\{a,b,c\right\}$.
3. Calculate the minimised mean error *E* between the sample *x* and the centre $\mu_{j}$, as shown in Equation (1):
  (1) $$E=\overset{k}{\underset{i=1}{\sum }}\underset{x\in C_{j}}{\sum }∥x_{i}-\mu_{j}∥{}_{2}^{2}$$
  In Equation (2), place the nearest $\mu_{j}$ to the corresponding cluster $C_{j}$ in the cluster, and repeat this process for the nearest $x_{i}$ in the cluster. Then, update $C_{j}$ = $C_{j}\cup \left\{x_{i}\right\}$.
4. Recalculate the new centre of mass
  (2) $$\mu_{j}=\frac{1}{\left|C_{i}\right|}\underset{x\in C_{j}}{\sum }x\;\;\;$$
5. If none the centres of mass have changed, the final cluster *C* = {., *k*}; otherwise, repeat steps 3 and 4 and iterate until the maximum number of iterations N is reached.

### 2.3. Bidirectional Long Short-Term Memory Networks

In RNNs, the problem of poor information persistence exists [18]. LSTM solves the problem of gradient explosion and gradient disappearance in RNNs by adding an adaptive forget gate that enables the release of internal resources and continuously performs dynamic updates during the learning process.

The LSTM neural network is passed backwards two pieces of data at a time, one in the normal hidden-layer state and one in the LSTM-specific hidden-layer state, which is passed as shown in the dashed box in Part A of the figure.

The LSTM also contains three gate structures [19], which are the forget gate, the input gate, and the output gate. These gate structures convert the input to a value between 0 and 1 through different activation functions, which are used to determine whether the information is retained or removed.

The result of one of the forget gates, which mainly determines whether the information can be discarded in preparation for the subsequent cell state update, is shown in the dashed box in Part B of the figure. Its equation is given as Equation (3):

(3) $$f_{t}=\sigma \left(W_{fO}O_{t-1}+W_{fx}X_{t}+b_{f}\right)$$

Equation (3) shows the weight coefficients for the current forget gate. The current input is multiplied by the weight coefficients, and the weight coefficients of the previous cell’s output are multiplied by the output. The sums of these values are added to the vector of the current cell as a bias term, and a range of values between [0,1] is output. The range of this result is determined by the activation function. The sigmoid function is generally chosen as the activation function [20], and the equation is as Equation (4).

(4) $$\sigma \left(z\right)=1/\left(1+e^{-z}\right)\;$$

The characteristics of the function are, among others, continuous derivability, gradient smoothing, and the ability to map real numbers directly into the [0,1] interval. The use of sigmoid as an activation function is justified from the basic role of forget gate in LSTM, as it acts as a saturating function that effectively remembers or forgets information, whereas unsaturated gates would always superimpose past and present information. The use of an unsaturated activation function would therefore lead to this superposition being exacerbated by subsequent use of the Bi-LSTM neural network, resulting in a straight line prediction.

Then, the value of the gate $i_{t}$ is given as Equation (5):

(5) $$i_{t}=\sigma \left(W_{iO}O_{t-1}+W_{ix}X_{t}+b_{i}\right)$$

where $W_{iO}$ and $W_{ix}$ are the weights of the inputs and are the bias terms. A new vector describing the current input is obtained by the nonlinear mapping of the sigmoid function here and finally by the tanh function, ss shown in Equation (6):

(6) $${\tilde{C}}_{t}=\tan h\left(W_{c}\left[h_{t-1},x_{t}\right]+b_{c}\right)$$

where tan*h*() is the hyperbolic tangent function.

The value of output gate $o_{t}$ is given as Equation (7):

(7) $$o_{t}=\sigma \left(W_{gO}O_{t-1}+W_{gx}X_{t}+b_{o}\right)$$

where $W_{gO}$ and $W_{gx}$ are the weights of the input items, and $b_{o}$ is the bias term. Then, the value of the memory cell at the current moment $C_{t}$ is calculated, as shown in Equation (8):

(8) $$C_{t}=f_{t}C_{t-1}+i_{t}{\tilde{C}}_{t}$$

The final output of the LSTM at the current moment can be calculated as Equation (9):

(9) $$h_{t}=o_{t}\tan h\left(C_{t}\right)$$

**Figure 3:**
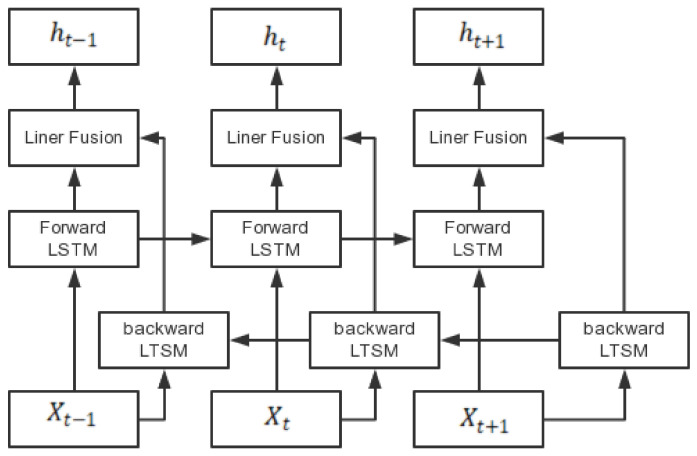
Bi-LSTM structure diagram.

The bidirectional long short-term memory network Bi-LSTM used in this paper was based on LSTM, which integrates the positive and negative information patterns of the temporal data, repeats the existing sequence of data with strong pre- and post-temporal correlations, and effectively extracts the temporal features in the signal. Bi-LSTM consists of two LSTM layers with the same number of neurons. Its structure is shown in Figure 3.

The Bi-LSTM first input the forward data into the forward LSTM layer to obtain the output of the forward LSTM layer. The data were then fed backwards into the inverse LSTM layer, and the output was reversed again to obtain the output of the inverse LSTM layer. Finally, the output of the forward LSTM layer was linearly superimposed on the output of the reverse LSTM layer to obtain the final output.

### 2.4. Construction of the Test Platform and Feature Extraction

#### Test Platform Construction

**Figure 4:**
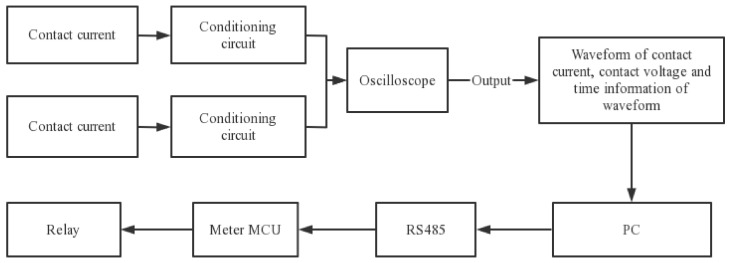
Block diagram of the test system.

**Figure 5:**
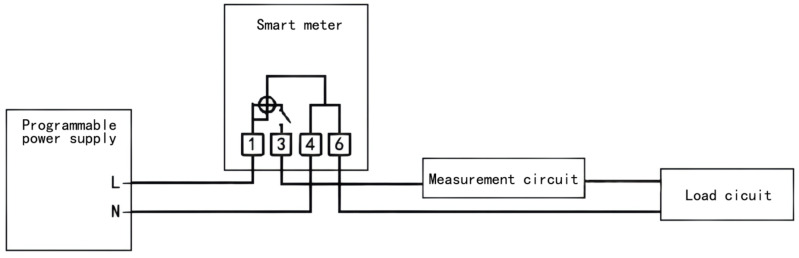
Schematic diagram of the circuit connections.

The meter relay test rig contained the following main components: contact current acquisition module, contact voltage acquisition module, relay control module, and oscilloscope, as shown in Figure 4. The circuit connections are shown in Figure 5.

The measurement circuit in the test bench mainly consisted of the contact current and voltage acquisition modules, where the current acquisition module mainly consisted of Hall sensors and conditioning circuits and the voltage circuit consisted of voltage divider resistors and conditioning resistors. The voltage circuit consisted of a voltage divider resistor and a conditioning resistor. The conditioning resistor was mainly composed of a filter circuit and an amplifier circuit. The programmable power supply in the diagram above was mainly used to output a specified current and power factor. To highlight the impact of the arc ignition energy on the relay, the test conditions were set to the inductive load conditions [21]. Therefore, a programmable power supply was required to regulate the circuit to the inductive load conditions to measure the arc ignition energy of the relay. The design test conditions are shown in Table 2 below.

## 3. Results

### 3.1. Feature Extraction

The arc energy of a relay is extracted by testing the voltage and current of the circuit during suction and closing. The arc energy is the discharge of the contact when the current is turned on and off, and is an important parameter in the study of the life of the appliance. As the arc energy grows, the erosion of contacts becomes more severe. Many studies show that the electrical erosion of electrical contact materials is directly proportional to the arc energy generated between contacts when the contacts are turned on and off, which is an important parameter reflecting the arc energy and contact surface erosion. Arc energy refers to the energy released during the arc ignition. Its value can be obtained by integrating the product of the arc voltage and current to the arc ignition time, so the arc energy Q generated from relay’s suction and closing is as shown in Equation (10):

(10) $$Q=\int_{t_{1}}^{t_{2}}u\left(t\right)i\left(t\right)\mathrm{dt}$$

where i(t) is the current at both ends of the contactor, u(t) is the voltage at both ends of the contact, t_1_ is the arc starting moment, and t_2_ is the arc extinguishing moment.

**Figure 6:**
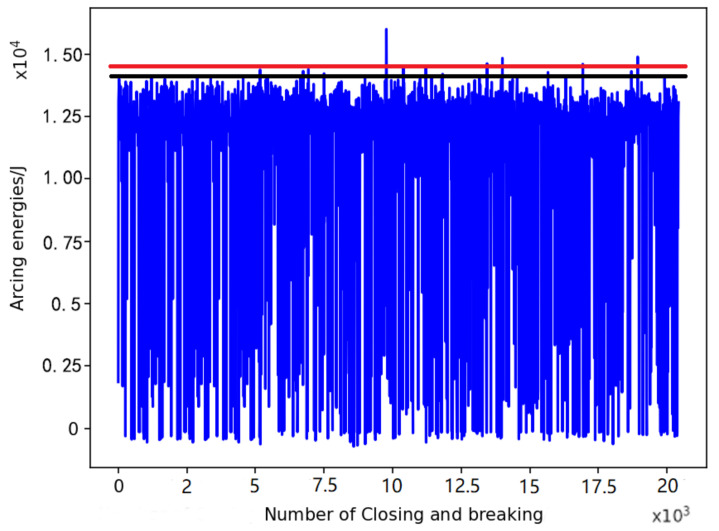
Arc ignition energy corresponding to closing and breaking.

First, the meter relay was tested to determine if there was a certain variation in the law. Then, the relay was opened and closed until its contact resistance reached 8 mΩ [22]. The relay is considered to have failed. The arcing energy was extracted by testing the voltage and current data of the circuit during the relay suction and closing processes, as shown in Figure 6.

From this figure, the low-risk area is set to 5000, which represents the maximum number of times the relay is opened and closed before the arc energy exceeds the black line threshold. When the number of times the relay is opened and closed is greater than 5000 but less than 7500, the arc energy exceeds the black line threshold more frequently and even reaches the red line threshold. This area is set as the medium-risk area. When the number of times the relay is opened and closed exceeds 10,000, the number of times the arc energy is greater than the black line threshold becomes more frequent, and the red line threshold is exceeded. This area is set as the high-risk area.

**Figure 7:**
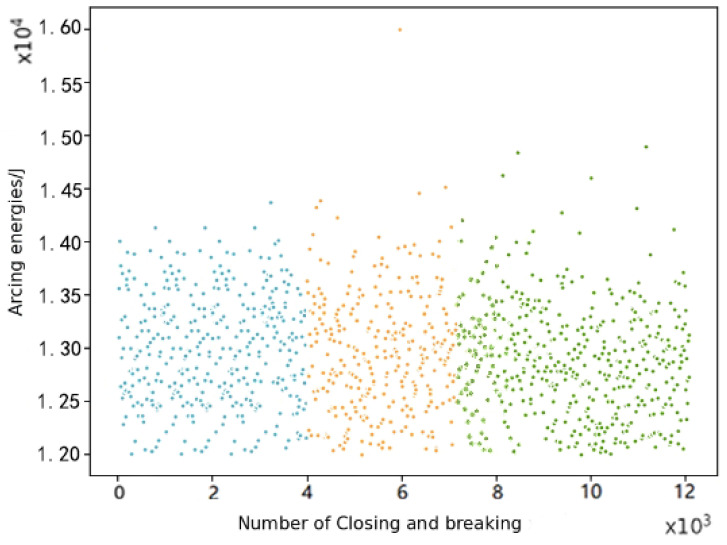
K-means clustering classification graph.

Therefore, the number of cluster centres in the K-means clustering method can be set to three, and as our focus is on the occurrence of high energy levels of arc energy, the data with arc energy less than 12,000 J are removed to enhance the feature-extraction capability when clustering. K-means clustering of the arc energy data was performed to obtain the results in Figure 7.

From this graph, we can see that higher energy levels of arc-burning energy occur more frequently between the different clustered regions. After clustering classification, we can obtain more scientific threshold values for each classification region using the actual classification results.

The average value of the peak in the blue low-risk area is set as the threshold L. If the frequency of exceeding threshold L is d, then the number of times the relay is opened and closed is the denominator, which is 4000 here, and the cumulative number of times the threshold L is exceeded based on 4000 relay openings and closings is the numerator *n*; Thus, the *d* is shown in Equation (11) below

(11) $$d=\frac{n}{4000}\cdot$$

**Figure 8:**
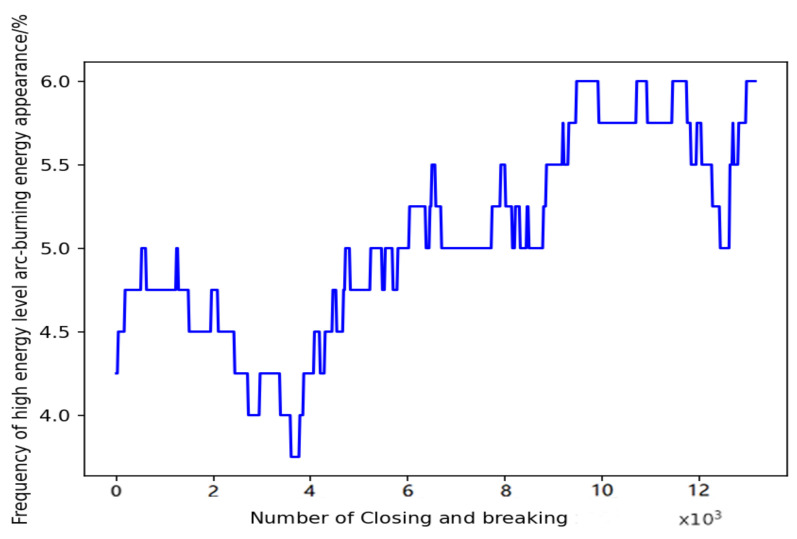
Frequency of high-level arc ignition energy corresponding to closing and breaking.

The frequency *d* in the green high-risk area in Figure 8 is then set as the failure threshold for reliability $L_{r}$, i.e., when the frequency of the meter relay exceeding the threshold L in 4000 relay openings and closings exceeds $L_{r}$, then the relay is considered to have failed. This is translated into a formalised arc energy degradation trend diagram, as follows below.

### 3.2. Model Building and Forecasting of Degradation Trends

#### Model Building

The input to the neural network in this paper is shown in the figure above, where the dataset based on the input training results is output. The following text is inserted to the chapter corresponding to the model establishment.

In this paper, the Bi-LSTM model is used to predict the degradation trend of the relays of meters. K-mean clustering algorithm is mainly used to implement the data type conversion of the arc-burning energy data as a characteristic parameter to describe the relay degradation. This is input to the Bi-LSTM model, and finally the prediction results of its degradation trend are output.

The arc-burning energy over time is first transformed into a dataset after the frequency data are transformed by the formula. The dataset then needs to be normalised to speed up the model calculation, enhance training convergence, and simplify the variability of the feature space. The normalisation is shown in Equation (12):

(12) $$X_{t}^{’}=\frac{X_{t}-X_{min}}{X_{max}-X_{min}}\cdot$$

The first 60% of the normalised dataset was used as the training set to train the Bi-LSTM model, the second 20% was used as the validation set to validate the training of the model, and the remaining 20% was used as a test set to judge how well the model was trained. Since the purpose of using deep learning in this paper was to perform predictive regression on trends in reliable lifetimes, the mean squared error (MSE) and goodness of fit $R^{2}$ were used to test the error between the predicted and true values of the model training set and the validation set. The MSE is given by Equation (13)

(13) $$\mathrm{MSE}=\frac{1}{N}\overset{N}{\underset{n=1}{\sum }}{\left(y_{n}-\hat{y_{n}}\right)}^{2\;\;\;\;\;\;\;\;}$$

where *N* is the number of samples and $y_{n}$ is the true value. The larger the value of MSE is, the worse the prediction.

(14) $$R^{2}=1-\frac{\sum {\left(\hat{y_{i}}-\bar{y_{i}}\right)}^{2}}{\sum {\left(y_{i}-\hat{y_{i}}\right)}^{2}}$$

As shown in Equation (14), the closer to 1 the value of $R^{2}$ is, the better the fit of the regression equation. $y_{i}$ is the true value, $\hat{y_{i}}$ denotes the output value in the deep learning model, and $\bar{y_{i}}\;$ is the average of true value.

### 3.3. Comparison of the Predicted Results and Outcomes of Degradation Trends

A comparison of LSTM and Bi-LSTM models for reliable life prediction of meter relays was made to determine the superiority of the methods. Figure 8 below shows the training data.

**Figure 9:**
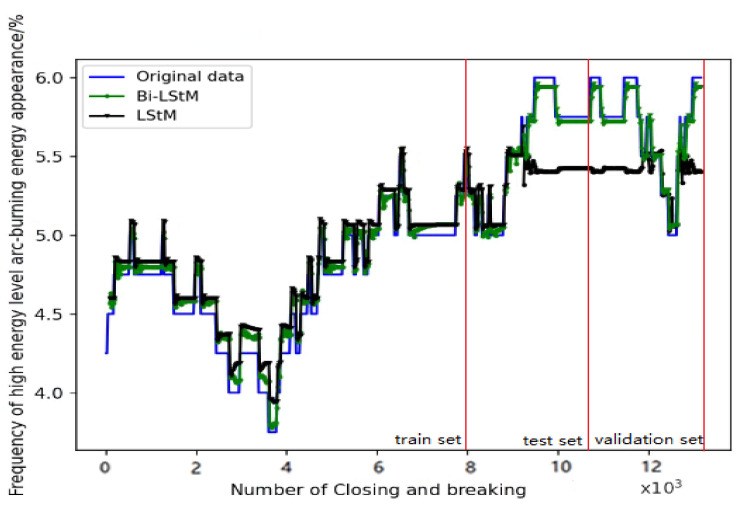
Plots of Bi-LSTM and LSTM predicted values fitted to the true values.

From Figure 9, we can see that the prediction accuracy of Bi-LSTM is higher than that of LSTM, especially in the test set and validation set, and the prediction is better than that of the LSTM neural network. From Table 3, we can see that the Bi-LSTM method is 12.31% more accurate than the LSTM method. This is due to the omission of some information in the one-way propagation of the LSTM, resulting in the prediction results deviating from the actual data. The bidirectional propagation in Bi-LSTM can improve this problem, while in the evaluation of the fitting accuracy, the MSE and prediction accuracy are 0.0016 and 0.067 higher than those of LSTM, respectively, indicating that the Bi-LSTM neural network performs better than LSTM in terms of fitting effect and prediction accuracy.

## 4. Conclusions

1. In this paper, a conversion of the form of arc-burning energy data is achieved by means of K-means cluster analysis. Arc-burning energy over time data are converted to a trend in the frequency of occurrence of high-energy level arc-burning energy over time. This enables the arc energy to be used to effectively describe the degradation trend of a relay without the need to disassemble the meter. The enhanced degradation characteristics of the arc energy greatly increase the accuracy of the subsequent neural network moaadel in predicting the degradation trend of the relay, and the effectiveness of using the arc energy to describe the degradation of the relay performance instead of the degradation parameters such as overtravel time and suction time is successfully verified.
2. The Bi-LSTM neural network model is used to achieve a more accurate prediction of the reliable lifetime. Its prediction accuracy is improved by 12.31% compared with LSTM, and the MSE and $R^{2}$ are 0.0016 and 0.067 higher than those of LSTM, respectively, indicating that the prediction effect and fitting accuracy of Bi-LSTM are better than LSTM under the conditions presented this paper.
3. The experimental results of this paper demonstrate that only the amount of data in the training set needs to be used, i.e., when performing reliability tests on meter relays, the number of openings and closings in the timed truncation test needs to be set to the same number as in the training set, and the predicted object is the same distribution of products under the condition that the degradation trend of the product can be better predicted to significantly reduce the test time. However, the disadvantage is that a larger number of relays need to be input into the first neural network construction to ensure that the products in the subsequent trials have approximately the same distribution as the input data in the first neural network construction.

The limitation of the method in this paper is that the method is only used to solve the arc energy such as the existence of special variation characteristic parameters of the data, meaning that it is not necessarily applicable to the trend prediction of other degradation parameters. On the other hand, a more comprehensive set of characteristic parameters of relay degradation requires the use of more degradation parameters to characterize relay degradation from different perspectives. Therefore, it is considered that multiple degradation parameters should be integrated in the next step for modelling to describe the degradation trend of relays more comprehensively.

## Figures and Tables

**Table 1:** Calculations of the main characteristic parameters of the relays.

Characteristic Parameter	Equation	Remarks
Release time	$t_{s}=t_{a}-t_{c}$	$t_{a}$ is the moment of arc generation, and $t_{c}$ is the moment when the coil is deenergised
Closing time	$t_{x}=t_{e}-t_{d}$	$t_{e}$ is the moment of contact of the relay, and $t_{d}$ is the moment when the coil is energised
Overtravel time	$t_{o}=$ $t_{b}-t_{f}$	$t_{b}$ is the moment of relay operation, i.e., the moment when the coil voltage rises, and $t_{f}$ is the point at which the coil current slopes to a positive value followed by a negative value
Arc-burning energy	$E=\frac{\sum_{n=a}^{b}u_{n}i_{n}}{f_{s}}$	$u_{n},i_{n}$ are the voltage and current of the contact, respectively, with a sampling range between the moment of suction and when the voltage change becomes smooth. $u_{n}$ is the point at 10–90% of the open circuit voltage. $f_{s}$ is the sampling rate.

**Table 2:** Test conditions.

Parameter	Value
Load voltage	220 V
Type of load	Inductive
Load current	65 A
Power factor	0.5 L
Rated current of the meter	60 A

**Table 3:** Comparison of the results of different algorithms.

Parameter	LSTM	Bi-LSTM
MSE	0.0126	0.0110
Average relative error	0.8562	0.9793
$R^{2}$	0.8801	0.9471

## Data Availability

Data will be provided upon request.
